# Second and Third Pandemic Waves in Apulia: How COVID-19 Affected Orthopedic and Trauma Care—A Single-Center Study

**DOI:** 10.3390/jcm11216526

**Published:** 2022-11-03

**Authors:** Giovanni Vicenti, Davide Bizzoca, Elisa Pesare, Michele Grasso, Walter Ginestra, Biagio Moretti

**Affiliations:** 1 Orthopaedics Unit, Department of Basic Medical Science, Neuroscience and Sensory Organs, School of Medicine, University of Bari “Aldo Moro”, AOU Consorziale Policlinico, 70124 Bari, Italy; 2 PhD Program in Public Health, Clinical Medicine and Oncology, University of Bari “Aldo Moro”, Piazza Giulio Cesare 11, 70124 Bari, Italy

**Keywords:** orthopedic surgery, trauma surgery, COVID-19, second wave, third wave, elective surgery

## Abstract

Purpose: In orthopedics and traumatology, as a direct consequence of the COVID-19 first wave, there was a massive reorganization and a stop to all elective activities, which were postponed. In this study, we aimed to analyze the impact of the COVID-19 pandemic on orthopedic surgery in Apulia during the second wave, from March to June 2021 (when Apulia was under social distancing restrictions), and during the third wave, from September to December 2021 (when Apulia was under no restrictions). We compared these months to the same periods in 2019 for an evaluation of the surgical decrease during the pandemic period. Methods: We performed a retrospective analysis of major orthopedic procedures, day-surgery procedures and urgent procedures (trauma and non-traumatic amputation) performed during the second and third waves of the pandemic in our clinic, and we compared these data with the same procedures performed in the corresponding periods of 2019, before the pandemic. Results: Surgical activity was significantly decreased during both periods; the only increase in surgical activity in 2021 compared to 2019 was in total hip, knee and shoulder arthroplasty, with a surge of +7.69% registered in the period September–December 2021. Conclusions: Longer waiting lists and limited healthcare resources were the big challenges for the orthopedic community, and they still represent a substantial issue to confront today.

## 1. Introduction

The COVID-19 pandemic had an unprecedented impact on our daily routine and surgical practice [1]. As the disease continued to spread, the high number of cases put an enormous burden on the healthcare system [2]. As a direct consequence, many elective procedures were cancelled, and all available resources were relocated to emergencies and COVID-19 [3,4].

In orthopedics and traumatology, there was a massive reorganization and a temporary stop to all elective activities, which were postponed. Urgent consultations were also cancelled to minimize the risk of infection for patients and medical staff [5]. 

On the contrary, outpatient activity remained open for fractures, surgery follow-up and orthopedic oncology cases [6,7].

However, while this process followed similar guidelines, it had significant regional variability [8]. In Apulia, the first pandemic wave was faced with the organization of a regional healthcare system and the first lockdown, which ended in June [9]. Throughout the following months, other pandemic waves broke out all around the world and even in Apulia, alternating with slowdown periods corresponding to late spring-summer months. Great efforts were made to safely resume elective procedures while still maintaining a safe medical environment for patients and hospital personnel [10,11].

During these new epidemic waves, elective surgical activity was heavily reduced but not completely abolished [10,12]. 

In this study, we aimed to analyze the impact of the COVID-19 pandemic on orthopedic surgery in Apulia during the second wave, from March to June 2021 (when Apulia was under social distancing restrictions), and the third wave, from September to December 2021 (when Apulia was not under restrictions). We compared these months to the same periods in 2019 for an evaluation of the surgical decrease during the pandemic period.

This study aims to quantify the profound impact of orthopedic elective surgery in our institution, which is the largest orthopedic center in Apulia, and share our experience of how this region is managing the post-outbreak period to date.

## 2. Materials and Methods

We performed a retrospective analysis of major orthopedic procedures, day-surgery procedures and urgent procedures (trauma and non-traumatic amputation) performed during our clinic’s second and third waves of the pandemic. We compared these data with the same procedures performed in the corresponding periods of 2019, before the pandemic.

Hospital admissions, main diagnoses and surgical procedures were retrieved from the electronic patient records and classified according to ICD-10 coding.

Elective procedures were defined as total hip and knee arthroplasty, revision joint replacement and arthroscopic surgeries for anterior cruciate ligament (ACL) reconstruction in patients admitted to the hospital. Spine surgeries were excluded from this analysis, since they differ in terms of surgical time and surgical complexity average admission days. Day-surgery procedures were defined as any arthroscopy for the meniscal repair of the knee, shoulder arthroscopy, tenolysis or neurolysis performed with outpatients, with no need for patient hospitalization for more than one day. Trauma admissions were defined as all hospital admissions with an ICD-10 code from the orthopedic trauma categories beginning with the letter “S” (injuries, poisoning and certain other consequences of external causes related to single body regions) and mainly including upper limb trauma, hip fractures and lower limb trauma. Elective and day-surgery procedures were performed after testing patients for COVID-19 negativity. Only negative patients were admitted to the COVID-free surgical area; on the other hand, traumatized patients were both COVID and non-COVID patients. COVID patients were not admitted to our orthopedic unit but were placed in specific COVID areas where our team could enter and surgically treat them. No distinction was made in this study between COVID and non-COVID cases. No data were collected and analyzed regarding the bed occupancy rate and the average admission day. A comparative descriptive analysis was performed using SPSS (version 23; IBM Corp, Armonk, NY, USA). The results of the quantitative variables were expressed as %.

## 3. Results

The results are presented as descriptive statistics. Data were analyzed to assess the variability between months in 2021 and the same period in 2019 in terms of surgery cases (planned and urgent surgery and day-service activities).

### 3.1. Elective Surgery 

Elective operations (articular prosthesis, cruciate ligament reconstruction, rotator cuff repair, arthrodesis, flatfeet, hallux valgus) fell by −83.47% from 236 in March–June 2019 to 39 in March–June 2021 and by −19.63% from 219 in September–December 2019 to 176 in September–December 2021 (Table 1).

Analysis of the elective operations showed a decrease in total hip, knee, shoulder arthroplasties and revision surgery of −78.10% from 110 in March–June 2019 to 24 in March–June 2021 and an increase of +7.69% from 130 in September–December 2019 to 140 in September–December 2021 (Table 1).

Knee arthroscopies decreased by −96.87% from 32 in March–June 2019 to 1 in March–June 2021 and by −75.00% from 32 in September–December 2019 to 8 in September–December 2021.

### 3.2. Day-Surgery Procedures 

Day-surgery procedures (implant removal, carpal tunnel syndrome, arthroscopy, trigger finger, neoformation removal) fell by −85.15% from 128 in March–June 2019 to 19 in March–June 2021 and by −61.94% from 155 in September–December 2019 to 59 in September–December 2021 (Table 2).

### 3.3. Trauma Surgery

The mean value of trauma operations fell by −62.91% in the four months from March to June 2021 compared to the same period in 2019 (135 vs. 364 total surgical procedures) and by −60.57% in the four months from September to December 2021 compared to the same period in 2019 (179 vs. 454 total surgical procedures).

#### 3.3.1. Lower Limb Trauma

The analysis of trauma surgery revealed that lower limb procedures were reduced by −71.85% from 199 in March–June 2019 to 56 in March–June 2021 and by −58.19% from 244 in September–December 2019 to 102 in September–December 2021 (Table 3). 

#### 3.3.2. Upper Limb Trauma

Upper limb trauma procedures showed a reduction of −56.29% from 151 in March–June 2019 to 66 in March–June 2021 and of −62.98% from 208 in September–December 2019 to 77 in September–December 2021 (Table 3).

### 3.4. Traumatic Amputations

During the lockdown period (March–June 2019), the traumatic amputations performed numbered 14, and in 2021, they numbered 13 (−7.14%). During the third wave (September–December 2019), the total number of amputations performed in the hospital was 15; on the contrary, from September to December 2021, the number increased to 22 (+46%) (Table 3).

## 4. Discussion

This quantitative study reports the full recovery of elective orthopedic surgeries in the age of COVID-19 in our Orthopedic and Trauma Unit in Policlinico di Bari, Apulia. We took two periods of 4 months each (March–June, September–December) to illustrate this achievement, comparing 2019 data (pre-COVID period) to 2021 data. The transition from the cessation of elective orthopedic surgeries to the recovery of pre-epidemic activity was analyzed. Elective surgery could restart with a lower risk of developing COVID-19 disease thanks to new protocols.

The full reopening of surgical activity in 2021 was slowed down by the hospital, aiming at blocking the spread of the virus and ensuring the safety of both patients and staff, protecting them with complete vaccination and with access to proper personal protective equipment (PPE). 

During the third wave, all our medical staff were more skilled than in the past in facing COVID-19 infections but also its consequences and effects on everyday clinical practice; the protocols changed very fast, but PPE was available (in contrast to the first pandemic period).

The reduction in elective surgery also reduced the workload for orthopedic surgeons during the second wave period, while, on the contrary, after restarting elective surgery, the work-hours balance was compromised by a very stressful work rate due to challenging waiting lists.

Reinstating orthopedic services in 2021 was aimed at unlocking the enormous pent-up demand for admissions and outstanding surgical procedures cancelled in the previous pandemic year. This objective was only partially achieved because of the reorganization of resources and anesthesiologists still being deployed to intensive care units to look after COVID patients.

In particular, in our hospital, a new COVID unit was set up at the expense of other departments; the beds in our unit were halved from 54 in 2019 to 27 in 2021. Some anesthesiologists previously assigned to our unit were relocated to the COVID unit as necessary at that moment, and this condition made a mandatory surgery prioritization strategy based on emergent/urgent/necessary patients much more necessary than in the past.

Furthermore, sometimes, it was the patients themselves who decided to decline orthopedic hospitalization because of the fear of contracting COVID-19.

All these reasons explain the high rate of surgical activity reduction.

The only increase in surgical activity in 2021 compared to 2019 was in total hip, knee and shoulder arthroplasty, with a surge of +7.69% registered in the period September–December 2021. In our opinion, these data are explicable, considering the great number of delayed surgeries, which accumulated with newly enlisted patients in 2021. Surgeons, under patient and waiting list pressures, hurried to reprogram all the cancelled surgical activity as far as the pandemic situation permitted. Furthermore, patients well accepted the modified return to elective surgery (the opportunity to create a COVID-free surgical area became possible thanks to new protocols, such as pre-operative COVID tests, vaccination status checks and screening for symptoms).

Day-surgery procedures (implant removal, carpal tunnel syndrome, arthroscopy, trigger finger, neoformation removal) fell by −85.15% in March–June 2019 compared to the same period in 2021 and by −61.94% in September–December 2019 compared to the same period in 2021 (Table 2). 

A complete stop to day-service activity in orthopedic surgery throughout the pandemic period was necessary, but comparing the number of procedures performed, it is possible to see a higher reduction in day-service activity during the second wave compared to the third wave reduction due to the very long waiting lists created by waiting patients in 2021.

Only traumatic amputations showed a different behavioural trend comparing the two periods analyzed; probably the most important reason was the worsening of chronic diseases [13] due to the stop of outpatient clinical visit follow-up. Patients affected by diabetic foot ulcers, circulatory disorders, infections and other chronic degenerative diseases could not be followed up with outpatient visits, and, as a consequence, they went through prolonged suffering from increased pain, reduced mobility and disease progression. They felt a negative impact even on their mental condition.

The conclusions of this study are very similar to what other European and American centers had experienced. A German review of 2021 asserts that in periods with rising numbers of COVID-19 infections, a drop in numbers of around 70% was noted [14,15,16].

In the periods of COVID case reduction, the number of elective surgeries reached values almost equal to previous years, and even a catch-up effect was noted.

An American study from 2020 confirmed an experience similar to ours. It aimed to quantify the volume of delayed THA and TKA procedures and projected the time it would have taken to care for these delayed patients. The results of these projections indicate that a substantial number of patients had surgery delayed, and it could take a considerable time for surgeons to catch up and restore optimal patient access to care [17,18,19].

This study has several limitations. In the first place, the data were retrieved from a single institution. However, we believe the results reflect the recovery of selective orthopedic surgery in the whole region because of the uniformity of regional policy. Moreover, our study did not contain all of the elective orthopedic surgeries but only the most common ones, which are more often performed in our clinic. Finally, the retrospective nature of the study is inevitable because of the nature of the research contents.

## 5. Conclusions

In conclusion, although orthopedics and trauma surgery do not appear to be on the front line of the pandemic experience, surgical activity decreased substantially during both periods of March–June 2021 and September–December 2021. The only increase in surgical activity in 2021 compared to 2019 was in total hip, knee and shoulder arthroplasty. The orthopedic and trauma unit required a re-organization of activity not only during the lockdown period (March–June 2021) but especially in the following months (September–December 2021) due to the need for flexibility in healthcare and surgery. Longer waiting lists and limited healthcare resources were the big challenges for the orthopedic community, and they still represent a substantial issue to confront today.

## Figures and Tables

**Table 1:** Elective surgery.

	Elective Surgery: March–June 2019	Elective Surgery: March–June 2021	VAR %
Tha, tka, tsa, revision	110	24	−78.10%
Rotator cuff repair	24	8	−66.7%
ACL reconstruction	16	5	−68.75%%
Arthrodesis tibiotalar joint, hallux valgus alignment, flat foot	86	2	−97.67%
Total	236	39	−83.47%
	**Elective Surgery:** **September–December 2019**	**Elective Surgery:** **September–December 2021**	**<**
Tha, tka, tsa revision	130	140	+7.69%
Rotator cuff repair	27	12	−55.5%
ACL reconstruction	19	16	−15.78%
Arthrodesis tibiotalar joint, hallux valgus alignment, flat foot	43	8	−81.39%
Total	219	176	−19.63%

ACL = anterior cruciate ligament.

**Table 2:** Day-surgery procedures.

	Day-Service Surgery: March–June 2019	Day-Service Surgery: March–June 2021	VAR %
Surgical hardware removal	45	4	−91.11%
Carpal tunnel syndrome	32	9	−71.87%
Arthroscopy and meniscal suture	32	1	−96.87%
Trigger finger	9	1	−88.89%
Biopsy and histopathology report	10	4	−60.00%
Total	128	19	−85.15%
	**Day-Service Surgery: September–December 2019**	**Day-Service Surgery: September–December 2021**	
Surgical hardware removal	78	19	−75.64%
Carpal tunnel syndrome	24	12	−50.00%
Arthroscopy and meniscal suture	32	8	−75.00%
Trigger finger	8	10	25.00%
Biopsy and histopathology report	13	8	−38.46%
Total	155	59	−61.94%

**Table 3:** Trauma surgery.

	TRAUMA Surgery: March–June 2019	TRAUMA Surgery: March–June 2021	VAR %
Upper limb fractures	151	66	−56.29%
Lower limb fractures	199	56	−71.85%
Traumatic amputation	14	13	−7.14%
Total	364	135	−62.91%
	**TRAUMA Surgery:** **September–December 2019**	**TRAUMA Surgery:** **September–December 2021**	**VAR %**
Upper limb fractures	208	77	−62.98%
Lower limb fractures	244	102	−58.19%
Traumatic amputation	15	22	46.67%
Total	454	179	−60.57%

## Data Availability

Not applicable.
